# CpG-oligodeoxynucleotides suppress the proliferation of A549 lung adenocarcinoma cells via toll-like receptor 9 signaling and upregulation of Runt-related transcription factor 3 expression

**DOI:** 10.3892/br.2014.257

**Published:** 2014-03-18

**Authors:** PRINCE AMOAH BARNIE, PAN ZHANG, PING LU, XIAOBO CHEN, ZHAOLIANG SU, SHENGJUN WANG, HUAXI XU

**Affiliations:** 1 Department of Immunology, School of Medical Science and Laboratory Medicine, Jiangsu University, Zhenjiang, Jiangsu 212013, P.R. China; 2 Division of Nephrology, Department of Internal Medicine, Affiliated People’s Hospital of Jiangsu University, Zhenjiang, Jiangsu 212001, P.R. China

**Keywords:** CpG-oligodeoxynucleotides, toll-like receptor 9, A549 cancer cells, Runt-related transcription factor 3, proliferation

## Abstract

The aim of the present study was to investigate the effect of CpG-oligodeoxynucelotides (CpG-ODN) on the proliferation of the A549 human lung adenocarcinoma cell line and the expression of Runt-related transcription factor 3 (Runx3) and investigate the association between the toll-like receptor 9 (TLR9) signaling pathway and Runx3 expression during A549 cell proliferation. Different concentrations of CpG-ODN were used in this study to stimulate A549 cells and the expression of Runx3 at the mRNA or protein level was detected by reverse transcription-polymerase chain reaction or western blot analysis. Moreover, Runx3 siRNA was synthesized and transiently transfected into the A549 cells and the MTT assay was used to detect the effects of CpG-ODN on transfected cell growth. Our data demonstrated that CpG-ODN significantly inhibited the proliferation of A549 cells. The expression of Runx3 in the mRNA and protein level was increased in A549 cells stimulated by CpG-ODN. The CpG-ODN-stimulated cell proliferation was significantly inhibited in Runx3 siRNA-transfected A549 cells. In conclusion, CpG-ODN may bind to TLR9, inhibit the proliferation of A549 cells and upregulate the expression of Runx3.

## Introduction

Toll-like receptors (TLRs) bind to microbe components by recognizing pathogen-associated molecular patterns, they activate cellular signal transduction pathways, stimulate innate immune responses and further adjust the adaptive immune system. TLRs are perceived as a bridge between innate and adaptive immunity. Research on TLRs has been mainly focused on inflammation, autoimmune disease and organ transplantation rejection; recently, the association between TLRs and tumorigenesis has attracted scientific attention (1).

Among TLRs, TLR9 is the sole family member for detecting DNA (2). TLR9 was originally identified as a sensor for bacterial DNA with abundant unmethylated CpG dinucleotides. However, mammalian DNA of self-origin, which has a low frequency of unmethylated CpG dinucleotides, may also stimulate TLR9. This is strongly underpinned by a recent study reporting that TLR9 recognizes the sugar backbone 2′-deoxyribose of DNA, but not its bases, suggesting the nucleotide sequence is not the primary target of TLR9 (2). Therefore, DNA released from damaged cells may trigger sterile inflammation via TLR9, acting as a damage-associated molecular pattern molecule.

Accumulating evidence demonstrates that TLR9, which is mainly expressed on immune cells, is also functionally expressed on lung cancer cells (1–4). TLR9 signaling may alter the biological character of lung cancer cells, including promoting proliferation and enhancing the metastatic potential of tumor cells, indicating that the activation of TLR signaling in lung cancer cells may contribute to the progression of lung cancer (3–5). The A549 human lung adenocarcinoma cell line highly expresses TLR9 and also exhibits positive expression of the Runt-related transcription factor 3 (Runx3) (6,7). Runx3, a novel tumor suppressor gene, was found to be downregulated in gastric, colon and lung cancer (8–12). CpG-ODN, being a TLR9 agonist, may rapidly stimulate T and B cells, induce Th1 cytokines [interleukin (IL)-1, IL-6, IL-12, IL-18, TNF-α and IFN-γ] and promote the maturation of antigen-presenting cells (APCs), indirectly activate immune cells and inhibit tumor proliferation. However, the association between the effect of CpG-ODN on lung cancer cells and Runx3 expression has not been determined. In this study, we aimed to elucidate the association between the TLR9 signaling pathway and Runx3 expression, laying the foundation for further invstigations on the antitumor mechanism of the TLR9 signaling pathway.

## Materials and methods

### Cell culture

The A549 human lung adenocarcinoma cell line was cultured in Dulbecco’s modified Eagle’s medium, supplemented with 100 U/ml penicillin, 100 mg/l streptomycin and 10% fetal bovine serum (Gibco-BRL, Carlsbad, CA, USA). In order to analyze the effect of TLR9 on cell proliferation, the cells were stimulated by CpG-ODN at different concentrations for 2, 4, 6 and 8 h and collected by centrifugation at 800 × g for 10 min at 4°C. Total RNA was isolated from cultured cells using an RNA extraction kit (Takara Bio, Inc., Shiga, Japan) and prepared for polymerase chain reaction (PCR) amplification.

### Reverse transcription-PCR (RT-PCR) and quantitative PCR (qPCR)

The cells were discharged into TRIzol reagent (Invitrogen Life Technologies, Carlsbad, CA, USA), total RNA was isolated by an RNA extraction kit (Takara) and reversed-transcribed with the ReverTra Ace^®^qPCR-RT kit (Toyobo, Osaka, Japan) according to the manufacturer’s instructions. The RT-PCR and qPCR were performed as previously described (13). The sequences for the primers used were as follows: β-actin (262 bp), 5′-CACGAAACTACCTTCAACTCC-3′ (forward) and 5′-CACGAAACTACCTTCAACTCC-3′ (reverse); Runx3 (353 bp), 5′-GATGGCAGGCAATGACGA-3′ (forward) and 5′-CATACTCCTGCTTGCTGATC-3′ (reverse). The relative quantification of mRNA expression was performed with the comparative threshold cycle method (8).

### Western blot analysis

The cells (1×10^6^) were washed with cold PBS and lysed in 100 μl lysis buffer [150 mmol/l NaCl, 20 mmol/l Tris-HCl (pH 7.5), 1 mmol/l EDTA, 1 mmol/l EGTA, 1 mmol/l Na_3_VO_4_, 1 mmol/l sodium fluoride, 0.5% DOC, 1% Triton X-100 and 1% Nonidet-P40]. The cell lysates were boiled with 2X loading buffer for 20 min and analyzed by western blotting. Mouse anti-human Runx3, mouse anti-human β-actin and anti-NF-κB were used as primary antibodies (Santa Cruz Biotechnology, Inc., Santa Cruz, CA, USA). Following polyacrylamide gel electrophoresis, the protein was transferred onto a PVDF membrane (Perkin-Elmer, Waltham, MA, USA). The membrane was incubated with the primary antibody, followed by incubation with horseradish peroxidase-conjugated rabbit anti-mouse IgG (Takara Bio, Inc., Shiga, Japan). After being thoroughly washed in Tris-Buffered Saline with Tween-20, the blots were processed for detection of Ag using an electrochemiluminescence plus western blotting detection system (GE Healthcare Life Sciences, Pittsburgh, PA, USA). The Typhoon Molecular Imaging system (GE Healthcare Life Sciences) was used for scanning and recording the results.

### Design of Runx3 siRNA

According to the design principle of RNA interference target sites, GCCACTTGATTCTGGAGGA was selected as the Runx3-specific sequence and synthesis of the dsRNA sequence was performed by Zimmer Medical Int’l Trading Co., Ltd. (Shanghai, China). The primers used were as follows: Runx3: sense, 5′-GCCACUUGAUUCUGGAGGATT-3′ and antisense, 5′-UCCUCCAGAAUCAAGUGGCTT-3′; control dsRNA, sense, 5′-UUCUCCGAACGUGUCACGUTT-3′ and antisense, 5′-ACGUGACACGUUCGGAGAATT-3′. All the sequences were labeled with fluorescence.

### Transfection of Runx3 siRNA

The A549 cells in logarithmic growth phase were cultured in 24-well plates and the transfection was performed when the cell confluence reached 80%, according to the manufacturer’s instructions (Lipofectamine^®^2000, Invitrogen Life Technologies, Carlsbad, CA, USA). The transfection efficiency was observed under a fluorescence microscope. The experiment included four groups: untransfected, transfection reagent control, negative sequence control and Runx3 siRNA-transfected groups.

### Proliferation assay

The cells were divided into A549, A549+CpG, A549-NC+CpG, A549-siRNA and A549-siRNA+CpG groups. A total of 1×10^6^ cells were seeded into 96-well plates; following cell adherence to the plate, 10 μl CpG were added to the wells at a concentration of 5 μl/ml and incubated at 37°C for 1~8 h. Subsequently, 20 μl (5 mg/ml) MTT were added to each well and the plate was further incubated for 4 h to deoxidize MTT under light-blocking conditions. After removal of the MTT dye solution, the cells were treated with 50 μl DMSO and the absorbance at 490 nm was measured using the EL×800 UV microplate reader (BioTek, Winooski, VT, USA). In each experiment and under each condition, proliferation was assessed in triplicate and the experiments were repeated at least twice.

### Statistical analysis

All the statistical analyses were performed using GraphPad Prism, software, version 5.0 GraphPad Software, San Diego, CA, USA). Data are expressed as means ± SD. Comparisons between groups were performed using the unpaired Student’s t-test. P<0.05 was considered to indicate a statistically significant difference.

## Results

### Expression of Runx3 in CpG-ODN-induced A549 cells

**Figure 1:**
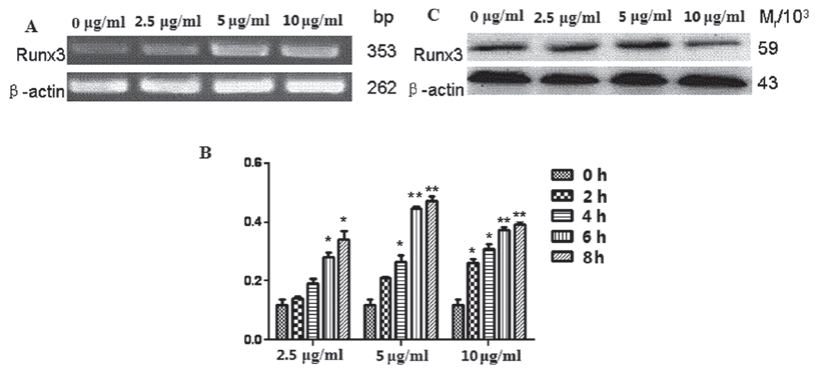
Expression of Runt-related transcription factor 3 (Runx3) in A549 cells stimulated by CpG. (A) Gel electrophoresis results of polymerase chain reaction (PCR) products. (B) The results of quantitative PCR demonstrated that the level of Runx3 mRNA was increased in A549 cells stimulated by CpG-ODN at different concentrations. (C) Expression level of Runx3 protein in A549 cells detected by western blot analysis. ^*^P<0.05 and ^**^P<0.01 vs. the control. M_r_/10^3^, relative molecular mass.

The analysis of the PCR amplification products by gel electrophoresis revealed a low expression level of Runx3 mRNA in A549 cells without CpG-ODN, which was increased after the cells were stimulated by CpG-ODN at concentrations of 2.5, 5 and 10 μg/ml for 2, 4, 6 and 8 h, respectively (Fig. 1). Similar results were obtained by qPCR. The expression of Runx3 mRNA was the highest in A549 cells stimulated by CpG-ODN at 5 μg/ml for 8 h, indicating a time-dependent effect (Fig. 1B). Furthermore, the western blot analysis results indicated that the expression level of Runx3 protein was consistent with the results of RT-PCR (Fig. 1C).

### Inhibitory effect of siRNA on Runx3 expression in A549 cells

**Figure 2:**
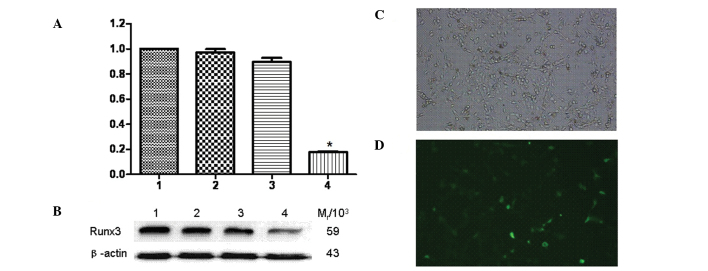
Expression of Runt-related transcription factor 3 (Runx3) mRNA in siRNA-transfected A549 cells. (A) The quantitative polymerase chain reaction results demonstrated that, compared to the untransfected group, the expression of Runx3 was inhibited after Runx3-siRNA was transfected into the A549 cells for 24 h. (Lanes: 1, untransfected cells; 2, transfection reagent control; 3, negative sequence control; and 4, Runx3 siRNA-transfected cells). (B) Results of the western blot analysis. Compared to the untransfected group, the Runx3 protein content was decreased significantly in Runx3 siRNA-transfected A549 cells. The relative molecular mass of protein Runx3 or β-actin (M_r_/10^3^) was 59 and 43, respectively. The Runx3 siRNA-transfected A549 cells were observed under (C) optical and (D) fluorescence microscope and the transfection efficiency was ~40%. ^*^P<0.05 vs. the control.

Runx3 siRNA-transfected A549 cells were observed under a fluorescence microscope and the proportion of fluorescent staining cells, representing the siRNA transfection rate, was 40% (Fig. 2C and D). The qPCR results demonstrated that, compared to the untransfected group, the expression of the Runx3 gene was inhibited in Runx3 siRNA-transfected A549 cells, but no significant difference was observed in untransfected cells (Fig. 2A). In addition, the western blot analysis revealed that the Runx3 protein expression was significantly decreased in transfected A549 cells compared to that in untransfected cells (Fig. 2B). The relative molecular mass of protein Runx3 (M_r_/10^3^) was 59 and that of β-actin was 43.

### Effect of Runx3 siRNA transfection on the proliferation of A549 cells stimulated by CpG-ODN

**Figure 3:**
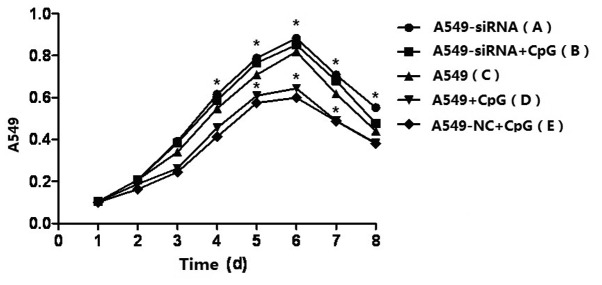
Growth curves of A549 cells detected by the MTT assay. As shown in the A549 cell growth curve, the proliferation was significantly inhibited by CpG-ODN stimulation of A549 cells (D), but there was no change in Runx3 siRNA-transfected A549 cells (B). There was a significant difference between the (B) and (C) groups (^*^P<0.05).

As shown by the cell growth curve (Fig. 3), the inhibition of cell proliferation following stimulation by CpG-ODN was markedly decreased in Runx3 siRNA-transfected A549 cells, almost to the original cell proliferation state. However, significant inhibition of A549 cell proliferation was observed in untrasfected A549 cells following CpG-ODN stimulation.

## Discussion

Runx3 is associated with the development and progression of gastric cancer and silencing of Runx3 in gastric cancer cells affects the expression of important genes involved in the metastatic process, including cell adhesion, proliferation and apoptosis; such silencing may promote peritoneal metastasis. The expression of Runx3 was found to be significantly reduced in human gastric mucosa exhibiting intestinal metaplasia and Runx3−/− mouse gastric epithelial cells bear the potential to differentiate into Cdx-2 positive intestinal-type cells (14).

CpG-ODN bind to TLR9 expressed on APCs, natural killer cells and other lymphocytes, mediating anti-infection or antitumor immune response, which has attracted significant attention among immunological investigators. Runx3 is a newly discovered tumor suppressor gene and its expression product may inhibit the proliferation of tumor cells through the transforming growth factor-beta (TGF-β) signaling pathway and induce apoptosis or maintain normal cell growth and development (15–17). The loss of Runx3 expression may lead to disorders of the TGF-β signaling pathway and is closely associated with tumorigenesis. Runx3, as a T-bet collaborative secondary transcription factor, is also involved in T- and B-lymphocyte differentiation and cytokine production (18). It was recently reported that CpG-ODN may directly upregulate the expression of T-bet in B cells by stimulating the TLR9 pathway as an alternative signal (19,20). In view of the dual role of Runx3, which inhibits the proliferation of tumor cells and regulates T and B cells, it is considered to be an important target for antitumor immunity.

In the present study, we investigated the expression level of TLR9 in the A549 cell line and observed the behavior of A549 cells stimulated by CpG-ODN. The results demonstrated that the CpG-ODN was able to significantly inhibit the proliferation of A549 cells and upregulate the expression of Runx3 in A549 cells at the transcriptional as well as the translational level. However, the expression level of Runx3 was decreased in Runx3 siRNA-transfected cells and the inhibitory effect of CpG stimulation on cell proliferation was distinctly reversed by Runx3 siRNA, indicating that CpG-ODN may inhibit the proliferation of TLR9-positive tumor cells by regulating the expression of the tumor suppressor gene Runx3 through the TLR9 signaling pathway. Furthermore, the upregulation of the Runx3 gene may also induce the expression of the transcription factor T-bet, promote Th1-type response and enhance antitumor immunity. It is hypothesized that the induction of Runx3 via the TLR9 signaling pathway may be of value in providing a wide range of therapeutic modalities and targets, which requires further investigation.

## References

[1] Chen R, Alvero AB, Silasi DA, Steffensen KD, Mor G (2008). Cancers take their Toll - the function and regulation of Toll-like receptors in cancer. Oncogene.

[2] Hazeki K, Uehara M, Nigorikawa K, Hazeki O (2013). PIKfyve regulates the endosomal localization of CpG oligodeoxynucleotides to elicit TLR9-dependent cellular responses. PLoS One.

[3] Schmausser B, Andrulis M, Endrich S, Muller-Hermelink HK, Eck M (2005). Toll-like receptors TLR4, TLR5 and TLR9 on gastric carcinoma cells: an implication for interaction with *Helicobacter pylori*. Int J Med Microbiol.

[4] Ren T, Wen ZK, Liu ZM, Liang YJ, Guo ZL, Xu L (2007). Functional expression of TLR9 is associated to the metastatic potential of human lung cancer cell: functional active role of TLR9 on tumor metastasis. Cancer Biol Ther.

[5] Chang YJ, Wu MS, Lin JT, Chen CC (2005). *Helicobacter pylori*-induced invasion and angiogenesis of gastric cells is mediated by cyclooxygenase-2 induction through TLR2/TLR9 and promoter regulation. J Immunol.

[6] Li QL, Kim HR, Kim WJ (2004). Transcriptional silencing of the RUNX3 gene by CpG hypermethylation is associated with lung cancer. Biochem Biophys Res Commun.

[7] Kato N, Tamura G, Fukase M, Shibuya H, Motoyama T (2003). Hypermethylation of the RUNX3 gene promoter in testicular yolk sac tumor of infants. Am J Pathol.

[8] Xu Y, Gao J, Su Z (2012). Downregulation of Hlx closely related to the decreased expressions of T-bet and Runx3 in patients with gastric cancer may be associated with a pathological event leading to the imbalance of Th1/Th2. Clin Dev Immunol.

[9] Kang KA, Kim KC, Bae SC, Hyun JW (2013). Oxidative stress induces proliferation of colorectal cancer cells by inhibiting RUNX3 and activating the Akt signaling pathway. Int J Oncol.

[10] Li M, Tan SY, Zhang J, You HX (2013). Effects of paeonol on intracellular calcium concentration and expression of RUNX3 in LoVo human colon cancer cells. Mol Med Rep.

[11] Lim J, Duong T, Do N, Do P, Kim J, Kim H, El-Rifai W, Ruley HE, Jo D (2013). Antitumor activity of cell-permeable RUNX3 protein in gastric cancer cells. Clin Cancer Res.

[12] Ito K (2012). Tumor suppressive functions of RUNX3 in gastric carcinogenesis. J Jpn Biochem Soc.

[13] Yang P, Qiu G, Wang S, Su Z, Chen J, Wang S, Kong F, Lu L, Ezaki T, Xu H (2010). The mutations of Th1 cell-specific T-box transcription factor may be associated with a predominant Th2 phenotype in gastric cancers. Int J Immunogenet.

[14] Yano T, Ito K, Fukamachi H, Chi XZ, Wee HJ, Inoue K, Ida H, Bouillet P, Strasser A, Bae SC, Ito Y (2006). The RUNX3 tumor suppressor upregulates Bim in gastric epithelial cells undergoing transforming growth factor beta-induced apoptosis. Mol Cell Biol.

[15] Fainaru O, Woolf E, Lotem J, Yarmus M, Brenner O, Goldenberg D, Negreanu V, Bernstein Y, Levanon D, Jung S, Groner Y (2004). Runx3 regulates mouse TGF-beta-mediated dendritic cell function and its absence results in airway inflammation. EMBO J.

[16] Li J, Kleeff J, Guweidhi A, Esposito I, Berberat PO, Giese T, Buchler MW, Friess H (2004). RUNX3 expression in primary and metastatic pancreatic cancer. J Clin Pathol.

[17] Miyazono K, Suzuki H, Imamura T (2003). Regulation of TGF-beta signaling and its roles in progression of tumors. Cancer Sci.

[18] Djuretic IM, Levanon D, Negreanu V, Groner Y, Rao A, Ansel KM (2007). Transcription factors T-bet and Runx3 cooperate to activate Ifng and silence Il4 in T helper type 1 cells. Nat Immunol.

[19] Liu N, Ohnishi N, Ni L, Akira S, Bacon KB (2003). CpG directly induces T-bet expression and inhibits IgG1 and IgE switching in B cells. Nat Immunol.

[20] Rosa R, Damiano V, Formisano L, Nappi L, Marciano R, Veneziani BM, De Placido S, Bianco R (2013). Combination of a Toll-like receptor 9 agonist with everolimus interferes with the growth and angiogenic activity of renal cell carcinoma. Oncoimmunology.

